# Factors associated with depression among young female migrants in Ethiopia

**DOI:** 10.1186/s12905-022-02017-0

**Published:** 2022-11-04

**Authors:** Annabel Erulkar, Girmay Medhin

**Affiliations:** 1 Population Council, P.O. Box 25562, Addis Ababa, Ethiopia; 2 Aklilu Lemma Institute of Pathobiology, Addis Ababa University, P.O. Box 1176, Addis Ababa, Ethiopia

**Keywords:** Mental health, Depression, Migrants, Commercial sex workers, Ethiopia

## Abstract

**Background:**

Mental health disorders represent a significant share of disease burden for adolescents and young people and depression is among the leading causes of morbidity within this age group. With rural-urban migration increasing in many settings, and young females being among the main migrants, few studies have examined the impact of such major transitions on mental health. This paper measures levels of depression among young women who are rural-urban migrants in Ethiopia, as well as factors associated with depression.

**Methods:**

This was part of a largescale study of urban migrant females aged 15–24 in Ethiopia, which took place in seven cities. Multiple categories of migrants were interviewed. We used modified PHQ-9 questions to measure depression and logistic regression models to examine its association with various characteristics including patterns of migration and violence. In all, 4,495 migrant females were interviewed.

**Results:**

Twenty-one percent of migrant young women displayed symptoms of moderate or severe depression. Symptoms of depression were more common among commercial sex workers (37%) than among other categories of migrants. Factors significantly associated with depression were being in commercial sex work (OR 1.70), migrating before age 15 (OR 1.37), using a broker to find a job (OR 1.53), experiencing forced first sex (OR 2.16) and experiencing beating in the last three months (OR 2.16).

**Conclusion:**

This study reveals significant levels of depression among young women in Ethiopia who are rural-urban migrants. The study highlights the need to expand measurement of mental health conditions in health surveys and underscores the need for additional investments in mental health infrastructure, programs and services for marginalized groups in sub-Saharan Africa.

## Introduction

Mental health disorders account for 13% of the global burden of disease, with depression being the third greatest contributor to the global disease burden [1]. Mental health disorders represent 16% of disease burden for adolescents aged 10 to 19—a comparatively larger share than other age groups—and depression is among the leading causes of morbidity within this age group [2]. A study conducted in 17 countries suggested that mental health disorders often begin during childhood or early adolescence (by age 14) and frequently remain undetected for decades [3].

The challenges related to prioritizing mental health are particularly glaring in sub-Saharan Africa. Mental health has received limited attention on the continent across multiple domains, including law and policy, health services and research. According to the World Health Organization’s (WHO) Mental Health Atlas 2017, among all the WHO regions, the African region countries report the lowest number of stand-alone mental health laws (44%), the lowest number of stand-alone policies (72%), the fewest number of mental health professionals (0.9 mental health workers per 100,000 population) and the fewest mental health facilities (0.1 facilities per 100,000) [4]. In addition, a review of papers published in *The Lancet Global Health* over a five-year period found that 637 papers were devoted to health issues in sub-Saharan Africa; among these, only 21 papers were devoted to depression, seven to anxiety and six to suicide [5]. Moreover, scant attention has been paid to mental health disorders among adolescents and youth in Africa, many of whom are at risk of mental health disorders as they face mounting pressures in the context of rapid population growth coupled with limited social and economic opportunities [5].

A meta-analysis of 12 studies of depression in Ethiopia found that, across the available studies, the pooled prevalence of depression was an estimated 11%, though methods of measurement and study populations varied across studies [6]. Many of the studies reviewed found that being female, being divorced or widowed and being a victim of intimate partner violence were consistently associated with a higher risk of depression and one study found that migrants are at three times greater risk of depression than non-migrants [6]. A more recent study in Northwest Ethiopia among nearly 800 adults estimated levels of depression at 18%, among which 11% exhibited mild symptoms, 4% moderate and 2% severe symptoms. In this study, females experienced elevated levels of depression compared to males [7]. Another study among out-patient adults in Sodo district Ethiopia found an estimated 12% had depression, with none of those screening positive for depression having the disorder detected during clinical consultation or treated [8].

Ethiopia is the second largest country in sub-Saharan Africa with an estimated population as of July 2020 of 108 million [9]. An estimated 42% of the population of Ethiopia are adolescents or youth. While rates of rural residence remain high in Ethiopia (80%), rates of urbanization are among the highest in sub-Saharan Africa, with an annual urban population increase of 4.63% [10]. In addition, the majority of internal, rural-urban migrants in Ethiopia are female. According to recent research by The World Bank, among rural migrants to Addis Ababa, 69% were female; among rural migrants to other urban areas of Ethiopia 56% were female [11]. The same study estimated that rural-urban migrants were aged 22 years, on average, making migrating populations markedly young and female.

This study examines the prevalence of depressive symptoms among Ethiopian girls and young women who migrated to urban areas. We also explore factors associated with depression, including demographic characteristics and the timing and pattern of migration.

## Methods

This is part of a larger study of out-of-school Ethiopian girls and young women aged 15 to 24, focusing on migration and the transition to different work roles following migration [12]. This was a large, mixed methods study that included formative qualitative research and a largescale quantitative survey. The quantitative study included questions to assess the mental health of those chosen for interview. The majority of survey respondents were rural-urban migrant females, but we also interviewed a small number of rural parents, rural girls and young women and brokers who help migrants find work. As the issue of mental health was not included in the initial qualitative research, we draw exclusively upon the findings from the quantitative study for the present study. Likewise, parent and broker interviews did not collect mental health information.

The quantitative study spanned six regions of Ethiopia and took place in seven cities: Adama, Addis Ababa, Dessie, Dire Dawe, Harar, Mekelle and Shashemene. These cities were chosen because they were considered to receive a large number of migrants from rural areas. Multiple categories of migrant girls and young women were interviewed. To be eligible for the study respondents had to be age 18 to 24, out-of-school and having left school by age 16, and to have migrated to the city before the age of 18. However, for domestic workers we included an age range of 15 to 24 in order to capture the experience of child domestic workers; at the same time, all our sampled domestic workers were above age 16. Most respondents were sampled through household listings, followed by random selection of eligible household members. Two additional categories of respondents were sampled purposefully: commercial sex workers and bar/café workers.

In each of the study cities, we identified neighborhoods where migrant young women were known to live, mainly low-income areas. These areas were identified based on consultation with local stakeholders and people knowledgeable about the areas, mainly government representatives. We conducted a systematic listing of all households and other structures in the area, such as back rooms of restaurants or establishments where young women may spend the night. Interviewers went house-to-house to list members of the household or structure, in order to identify girls and young women who were eligible for the study. Eligible females were sampled randomly, using the random number generator available in SPSS v.25.

In order to assess respondents by occupation, we asked (1) What type of work for pay have you done in the last three months? and (2) Of these, which is your main source of cash income and/or in-kind payment? The second questions was included in cases where respondents were engaged in more than one type of paid work. Commercial sex workers and bar/café workers are more hidden and stigmatized populations. Bar/café workers are often considered to partially engage in commercial sex work, which results in stigma. These categories of respondents were sampled purposefully, visiting places where targeted respondents were known to congregate (bars, cafes, nightclubs, local brew houses and red-light districts) and approaching potential respondents working in the bar/café or working as commercial sex workers for interview. Once contact was made, interviewers screened respondents for eligibility such as being migrant to the area and being out-of-school.

Survey interviewers were recruited from the study cities to ensure that they possessed relevant language capabilities and understanding of the local culture and community. Our female sample was interviewed by only female interviewers. The survey interviewer training lasted seven days. Interviewers reviewed the questionnaire item-by-item, reviewed ethical procedures including informed consent and actions to be taken in the case of adverse events, and engaged in practice interviews in pairs, ensuring understanding and adherence to skip patterns and general questionnaire administration. A professional counselling firm was also made available to respondents in case they had negative reactions based on the interview or expressed a need for such services.

A structured questionnaire was developed, pretested and translated into local languages Amharic, Oromiffa and Tigrigna. The pretest was undertaken in selected study cities and in locations outside of the study areas. Questionnaires elicited information on demographic and socioeconomic characteristics, education, families and social networks, migration, livelihoods, use of job placement brokers in finding work, mental health, marriage, sexual experience, HIV knowledge and behavior, family planning and pregnancy, and utilization of services. All data was transported from the field and entered in Addis Ababa by trained data entry clerks. Data was converted to SPSS v.25 for analysis. The study received ethical approval from the Population Council’s Institutional Review Board (IRB) and the National Research Ethics Committee in Ethiopia.

### Measures

In order to measure symptoms of depression among respondents, we used a modified patient health questionnaire (PHQ-9), asking about symptoms experienced in the two weeks prior to survey. PHQ-9 was developed to assess depression in primary care settings [12]. Several facility-based studies have validated PHQ-9 in Ethiopia [13, 14]. In the current study, respondents were asked the following: “I will read you a list of feeling or experiences one may have. I would like you to tell me if you have experienced this over the last two weeks: 1) Little interest or pleasure in doing daily activities, 2) Feeling down, depressed or hopeless, 3) Feeling tired or having little energy, etc. If the respondent answered ‘yes’ to any item, they were asked a follow-up question about how frequent the experience was: occasionally, on several days or daily. Three statements used in the standard PHQ-9 battery of questions were separated into two items. This is because, during pretest of the questionnaire, respondents found some of the statements confusing, overly complex, or difficult to understand. Compared to previous studies that validated PHQ-9 questions, this observed confusion may be due to the young age or low level of education of our study population. Questions separated into two items were (separation denoted by a. and b.): 1) a. Trouble falling asleep/staying asleep or b. sleeping too much, 2) a. Poor appetite or b. overeating, 3) a. Moving or speaking so slowly that other people could have noticed or b. being so fidgety or restless and move around more than usual so that other people have noticed. During analysis the separated items were back-coded so that the responses fit back to the original PHQ-9 and the total scores ranged from 0 to 27. Each item was scored from 0 to 3 to reflect the existence and frequency of symptoms. An overall score of 0-4 on PHQ-9 was considered no depression, those who scored 5–9 were coded has displaying moderate depression; those with a score of 10 or over were considered to have severe depression [14].

We present the percentage of young women in each category (domestic workers, commercial sex workers, waitresses/bar workers, other occupational categories and those not working) who have moderate or severe depression. We also examine the association between depression and various demographic characteristics as well as the experience of social isolation, patterns of migration and violence. Logistic regression was used to model the odds of experiencing moderate or severe depression. We assessed multicollinearity of the independent variables using ViF, with any value more than 10 indicating the existence of multicollinearity. We also assessed any potential effect of outliers using Cooks Distance. Variables with significant associations in the bivariate analysis were found to be uncorrelated, except for age and marital status which had a weak correlation.

In addition to basic demographic variables such as age, religion and marital status, we explored the association between depression and aspects of girls’ migration and social life. We included measures of early age at migration (below age 15) and having migrated on one’s own, without accompaniment of family members or other acquaintances. In addition, many migrating girls and young women use brokers in the course of migration to assist in securing them jobs. Council research has also demonstrated that some brokers may take advantage of migrating girls, which can result in sexual abuse and putting girls at increased risk for trafficking [15]. We included a measure of social connections and social isolation through the variable reflecting whether or not the respondent reports having friends. Finally, we also included in the model measures of violence such has having been beaten in the last three months and have experienced coerced or forced sexual initiation. Forced sexual initiation was calculated as the percent of girls who experienced first sex through any form of force or coercion, including being physically forced to have sex; their partner using violence to have sex; being locked in a room to have sex against her will; or their partner not taking “no” for an answer. Finally, because commercial sex workers had much higher levels of depression than other occupational categories, we included a covariate reflecting if the respondent was a commercial sex worker.

## Results

A total of 4,495 migrant females were interviewed in seven cities. Table 1 shows the characteristics of the sample of migrant girls and young women, by major occupational groups. On average, sampled girls were age 20, with over half (54%) being Orthodox Christian, 36% were Muslim, and 10% followed another religion. Respondents across categories had extremely low levels of education, on average below five years of schooling (mean 4.2 years of education). Domestic workers had the fewest average years of schooling (mean 3.7), followed by commercial sex workers (mean 4.1). Nearly one quarter of migrant girls had never attended school (24%). About 1 in 10 respondents (9%) were double orphans, with commercial sex workers reporting higher rates of double orphanhood (17%) compared to the other categories of respondents. Regardless, a very small proportion of respondents lived with at least one parent (3%).

**Table 1:** Sample characteristics, by current occupation of the respondent

	Not working (n = 1,607)	Domestic workers (n = 1,141)	Commercial sex workers (n = 796)	Waitress/ Bar workers (n = 510)	Other occupations (n = 441)	All urban migrants (n = 4,495)
**Age (mean)**	20.5	19.3	20.8	20.0	20.4	20.2
**Religion**						
Orthodox Christian	39.4	59.5	67.2	68.7	53.4	54.1
Muslim	50.6	31.3	22.2	21.0	33.2	35.6
Other	10.0	9.2	10.6	10.3	13.4	10.3
**Years of education (mean) Educational attainment**	4.2	3.7	4.1	5.3	4.0	4.2
None	22.2	26.1	28.8	12.9	26.8	23.7
1 to 4 years	28.1	32.0	19.2	20.6	24.9	26.3
5 to 8 years	44.3	39.2	48.5	59.0	42.0	45.2
9 + years	5.4	2.7	3.5	7.5	6.3	4.8
**Number of living parents**						
None	8.9	4.9	17.3	7.5	8.0	9.1
One parent	27.9	25.1	31.1	24.4	32.0	27.8
Two parents	63.2	70.0	51.6	68.1	60.0	63.1
**Live with parent(s)**	5.0	2.3	1.1	3.3	4.8	3.4
**Marital status**						
Never married	20.0	79.1	69.6	71.5	47.6	52.3
Currently married	76.2	11.4	5.5	17.3	41.7	37.2
Formerly married	3.8	9.5	24.8	11.2	10.7	10.5
**Migrated before age 15**	44.1	34.1	31.7	31.9	46.9	38.3
**Migrated alone**	29.7	35.0	59.6	50.3	37.6	39.5
**Asset ownership**						
Owns mobile phone	62.9	53.4	70.5	77.3	77.1	64.8
Owns a blanket	67.1	42.6	55.7	58.2	64.9	57.6
Owns bed	22.2	6.7	23.2	18.8	22.7	18.1
Owns a radio	16.2	3.9	15.5	13.5	14.3	12.5
**Percent with 2 + assets (above)**	53.0	25.1	51.2	53.3	56.9	46.1

Compared to other respondent categories, commercial sex workers were more likely to be formerly married (divorced, widowed, separated) (25%), compared to domestic workers (10%), waitress and bar workers as well as those in other occupations (11%, each). Young women who were unemployed or not working were much more likely to report being currently married (76%) compared to the other occupation groups. When asked about the context of migration, a relatively high proportion of young women migrated on their own (40%), with 60% of commercial sex workers making this transition on their own.

Measurement of household socio-economic status is a challenge, given the differing circumstances in which respondents live. For example, domestic workers frequently live with their employers, meaning measuring household assets would not be a reflection of a domestic workers’ own economic status. As such, we measure a limited number of personal assets in an attempt to reflect individual economic status. We asked respondents about ownership of different personal assets, such as a radio, mobile phone, bed and blanket. Nearly two-thirds (65%) of respondents owned a mobile phone and 58% owned a blanket; only a minority owned a bed (18%) or radio (12%). We calculated the percent of respondents who owned at least two of the four assets mentioned. Roughly half of the respondents in all categories owned at least two of the assets named with the exception of domestic workers, among whom only 23% owned two or more of the mentioned assets. This could reflect lower economic status among domestic workers or that some assets, such as blankets and beds, were being provided by their employer.

Using PHQ-9 to measure symptoms of depression, one in five migrant girls (21%) displayed symptoms of depression, whether moderate or severe (Table 2). Nearly 5% reflected symptoms of severe depression. Depressive symptoms were much more frequent among commercial sex workers than the other categories of respondents. Over one third of commercial sex workers (37%) displayed symptoms of depression (moderate or severe) and 10% reflected severe depressive symptoms.

**Table 2:** Percent of migrant adolescent girls and young women with symptoms of depression, by current occupation of the respondent

	Not working (n = 1,607)	Domestic workers (n = 1,141)	Commercial sex workers (n = 796)	Waitress/ Bar workers (n = 510)	Other occupations (n = 441)	All urban migrants (n = 4,495)
**No depression**	82.5	84.1	63.3	80.8	77.8	78.9
**Moderate depression**	14.7	12.4	26.9	14.7	15.9	16.4
**Severe depression**	2.7	3.5	9.8	4.5	6.3	4.7
**Any depression (moderate or severe)**	17.4	15.9	36.7	19.2	22.2	21.1

Table 3 shows bivariate associations between the experience of depression in the last two weeks and various background and social characteristics. The experience of depression is significantly associated with age, religion, marital status, being a single or double orphan, migrating at a young age, migrating alone/unaccompanied, using broker(s) for job placement, experiences of being beaten in the last three months, experiencing non-consensual first sex and being a commercial sex worker.

**Table 3:** Percent of migrant adolescent girls and young women with moderate or severe depression, by background characteristics

CHARACTERISTICS	No depression (n = 3,577)	Moderate or severe depression (n = 956)	P value
**Age**			**< 0.002**
15 to 19	45.1	39.3	
20 to 24	54.9	60.7	
**Religion**			
Orthodox Christian	52.7	59.8	**< 0.001**
Muslim	36.7	30.6	
Other	10.5	9.6	
**Education**			**NS**
< 5 years education	50.2	49.6	
5 + years education	49.8	50.4	
**Marital status**			**< 0.001**
Never married	52.4	51.6	
Currently married	38.7	32.5	
Formerly married	9.0	15.9	
**Orphan (single or double)**	35.4	42.0	**< 0.001**
**Migrated before age 15**	37.0	43.2	**< 0.001**
**Migrated alone/unaccompanied**	38.0	44.9	**< 0.001**
**Has no friends**	49.6	48.1	**NS**
**Ever used a broker**	26.8	41.3	**< 0.001**
**Beaten in last 3 months**	4.6	13.0	**< 0.001**
**Forced sexual initiation**	13.0	29.2	**< 0.001**
**Current occupation commercial sex worker**	14.2	30.7	**< 0.001**

Table 4 shows summary results from multivariable modelling where the outcome is depressive symptoms among migrant females. Our data do not show any evidence of multicollinearity as none of the VIF values were greater than 10; as well, there were no obvious influential observations, as evidenced by Cook’s Distance. Being a commercial sex worker (OR 1.7), migrating before the age of 15 (OR 1.4), and using a broker to find a job (OR 1.5) were all associated with increased odds of having depression. Variables reflecting the experience of violence were most strongly associated with the increased odds of having depression, with the odds of being depressed among those having been beaten 1.9 times compared to those who had not; the odds of having depression among respondents who experienced forced/coerced first sex were 2.2 times higher compared to than those who had not.

**Table 4:** Adjusted odds ratios (and 95% confidence intervals) from logistic regression analysis to identify associations between selected characteristic of female migrants and risk of depression

	Has depression (n = 4,533)	P value
**Age category**		
15–19 (Ref)	-	-
20–24	1.069 (0.905–1.262)	NS
**Religion**		
Orthodox Christian	-	
Muslim	0.882 (0.744–1.046)	NS
Other	0.860 (0.662–1.117)	NS
**Marital status**		
Never married (ref)	-	-
Currently married	1.014 (0.830–1.225)	NS
Formerly married	1.255 (0.985-1.600)	NS
**Occupation commercial sex worker**	1.699 (1.382–2.089)	p < 0.001
**Migrated before age 15**	1.374 (1.175–1.606)	p < 0.001
**Migrated alone/unaccompanied**	1.149 (0.980–1.347)	NS
**Ever used a broker to find a job**	1.525 (1.296–1.793)	p < 0.001
**Beaten (last three months)**	1.911 (1.458–2.506)	p < 0.001
**Forced sexual initiation**	2.162 (1.796–2.603)	P < 0.001

## Discussion

We conducted a largescale study of migrant girls and young women in Ethiopia which applied a standardized, tested and widely accepted method for measuring depression. To our knowledge, it is one of the few studies to examine the mental health of adolescent girls and young women in sub-Saharan Africa. We understand the study has limitations. Our data are cross-sectional and, therefore, do not establish causality.  In addition,  our sample of sex workers and bar/café workers was not random, which has potential for bias and violation of assumptions behind inferential tests.

Previous studies of depression in Ethiopia reflected a pooled prevalence of depression of roughly 11%, with depression associated with being female, formerly married and experiencing gender-based violence [6]. Compared to these earlier studies in Ethiopia across various populations, this study found higher estimated prevalence of depression among migrating girls and young women. Thirty-seven percent of girls and young women in commercial sex work reported symptoms of depression, whereas respondents in other occupation categories ranged from 16 to 22% exhibiting symptoms of depression. Data from the overall report of this study demonstrate that many girls and young women undertake migration with very little planning, preparation or awareness of what awaits them in urban centers [16]. This lack of preparedness and support, and probable hardships faced upon arrival, may result in the increased likelihood of depression among migrating girls, especially when doing so in isolation or at a young age. Indeed, migrating before age 15 was significantly associated with depression. Early migration may reflect a level of isolation, marginalization from one’s family and community and desperation among young migrant women who are compelled to make a major physical relocation at a young age.

Contact with job placement brokers was also significantly associated with elevated levels of depression. Research is emerging that job placement brokers who assist arriving migrant girls to find jobs in the city may not only be a source of support, but also result in sexual abuse and trafficking into commercial sex work/commercial sexual exploitation [15]. Focused research and programmatic attention should be devoted to the role that job placement brokers play in the migration experience of girls and young women, whether positive or negative, as little is known about the role they play in the migration transition.

This study highlights significant levels of depression among girls and young women in Ethiopia who are rural-urban migrants. As a population, these girls and young women have limited social support and social networks, little or no education, and limited opportunities for safe and rewarding livelihoods. After migration, many are absorbed into low-status work roles and a considerable number transitioned between different forms of low-status, exploitive and potentially dangerous jobs, such as domestic work and commercial sex work. The study highlights the need to expand measurement of mental health conditions in health surveys. Importantly, our results underscore to need for additional research as well as critical investments in mental health infrastructure, programs and services for marginalized groups in sub-Saharan Africa.

## Data Availability

The dataset used and/or analyzed during the current study are available from the corresponding author on reasonable request.

## List of abbreviations

IRB: institutional Review Board
HIV: human Immunodeficiency Virus
PHQ-9: patient Health Questionnaire
WHO: world Health Organization

## References

[1] Collins P, Patel V, Joestl S, et al. Grand challenges in global mental health. Nature 2011: Volume 475, July. DOI: 10.1038/475027a. 10.1038/475027a PMC3173804 21734685

[2] World Health Organization (WHO). Adolescent mental health. 2019. Geneva: WHO fact sheets, Available at https://www.who.int/news-room/fact-sheets/detail/adolescent-mental-health, Accessed January 28, 2020.

[3] Kessler RC, Angermeyer M, Anthony JC, et al. Lifetime prevalence and age-of-onset distributions of mental health disorders in the World Health Organization’s World Mental Health Survey Initiative, World Psychiatry. 2007; 6: 168 – 76. PMC2174588 18188442

[4] World Health Organization (WHO). Mental health atlas 2017. Geneva 2018: License CC BY-NC-SA 3.0 IGO.

[5] Sankoh O, Sevalie S, Weston M. Mental health in Africa. Lancet Glob Health. 2018 Sep;6(9):e954-e955. doi: 10.1016/S2214-109X(18)30303-6. PMID: 30103990. 10.1016/S2214-109X(18)30303-6 30103990

[6] Bitew T (2014). Prevalence and risk factors of depression in Ethiopia: a review. Ethiop J Health Sci.

[7] Molla GL, Sebhat HM, Hussen ZN. et. al. Depression among Ethiopian Adults: Cross-Sectional Study. Psychiatry J 2016, 1–5. doi:10.1155/2016/1468120. 10.1155/2016/1468120 PMC4876238 27247932

[8] Rathod SD, Roberts T, Medhin G (2018). Detection and treatment initiation for depress and alcohol use disorders: Facility-based cross sectional studies in five low-income and middle-income country districts. BMJ Open.

[9] CIA. 2020. The World Factbook: Ethiopia, Available at https://www.cia.gov/library/publications/the-world-factbook/geos/print_et.html, Accessed October 8, 2020.

[10] United Nations, Department of Economic and Social Affairs, Population Division. 2018. World Urbanization Prospects: The 2018 Revision, custom data acquired via website.

[11] Bundervoet T (2018). Internal Migration in Ethiopia: Evidence from a Quantitative and Qualitative Research Study.

[12] Erulkar A, Medhin G, Negeri L. The Journey of Out-of-School Girls in Ethiopia: Examining Migration, Livelihoods and HIV. Addis Ababa: Population Council, 2017.

[13] Kroenke K, Spitzer RL, Williams JB (2001). The PHQ-9: validity of a brief depression severity measure. J Gen Intern Med.

[14] Gelaye B, Williams MA, Lemma S, et al. Validity of the Patient Health Questionnaire-9 for depression screening and diagnosis in East Africa. Psychiatry Res. 2013;210(2):653-661. doi:10.1016/j.psychres.2013.07.015 10.1016/j.psychres.2013.07.015 PMC3818385 23972787

[15] Hanlon C, Medhin G, Selamu M. et. al. Validity of brief screening questionnaires to detect depression in primary care in Ethiopia. J Affect Disord. 2015 Nov 1;186:32 – 9. doi: 10.1016/j.jad.2015.07.015. Epub 2015 Jul 21. PMID: 26226431. 10.1016/j.jad.2015.07.015 26226431

[16] Erulkar A (2020). Characteristics of job placement brokers in relation to migrating girls and young women in Ethiopia.

